# When colleague got recognized: Third-party’s reaction to witnessing employee recognition

**DOI:** 10.3389/fpsyg.2023.968782

**Published:** 2023-03-27

**Authors:** Tianfei Yang, Xia Jiang

**Affiliations:** ^1^Business School, Ningbo University, Ningbo, China; ^2^College of Economics and Management, China-Africa International Business School, Zhejiang Normal University, Jinhua, China

**Keywords:** employee recognition, perceived organizational justice, workplace wellbeing, work engagement, chain-mediating effect

## Abstract

Employee recognition, an incentive method widely used in management practice, plays an important role in the organization. Although extant studies have confirmed its effectiveness, little attention has been paid to its spillover effect. Based on the Social Cognitive Theory and Affective Events Theory, this study argues that employee recognition encounter can trigger cognitive and behavioral reactions. Through perceived organizational justice and workplace wellbeing, a chain-mediating effect connects witnessing employee recognition to work engagement. This research adopts the empirical research method by asking the participants to do the weekly survey (four times in 1 month), and 258 samples are collected. Using SPSS20.0 and its PROCESS macro module, hypotheses are tested. The results indicate (1) employees who witness leaders’ recognition of colleagues will (a) perceive higher organizational justice and (b) be more engaged in work. (2) Perceived organizational justice will mediate the positive relationship between employee recognition encounters with (a) workplace wellbeing and (b) work engagement. (3) Perceived organizational justice and workplace wellbeing will have a chain-mediating effect in the path of employee recognition encounter and work engagement. The results provide both practical and theoretical contribution to employee recognition.

## 1. Introduction

Employee recognition is a popular approach to achieving leadership effectiveness, which is widely used in contemporary human resource management (Luthans, 2000). Employee recognition is a constructive feedback behavior adopted based on employees’ value judgments, including work performance, work commitment, and dedication (Brun and Dugas, 2008). Some scholars highlight that recognition is essentially an important vehicle for motivation (van Woerkom and Kroon, 2020). Workplace recognition may come from colleagues, subordinates, and supervisors (Brun and Dugas, 2008). In this study, we focus on the recognition of superiors in particular, which we believe is very important because it can guide employees to understand whether the organization recognizes their values (Eisenberger et al., 2010). In fact, because of the authority of their superiors and the particularity of their positions, they are empowered to provide recognition to employees and to recognize the contributions that individuals have made to the organization (Cannon, 2015). Three-quarters of organizations use formal recognition methods to motivate employees in practice (Garr, 2012), such as selecting “Excellent Employee of the Month” and “Star of the Week.” Compared with other motivational techniques, employee recognition has unique advantages. It is mostly non-material and symbolic appreciation or rewards (Montani et al., 2020) that can improve employees’ work performance at very low financial costs (Mosley and Irvine, 2014). Many studies have examined the role of recognition on employee outcomes, such as performance (Magnus, 1981; Godkin et al., 2010), individual wellbeing (Warr, 1987; Cropanzano and Mitchell, 2005; Gilbert and Kelloway, 2018), organizational citizenship behaviors (Cropanzano and Mitchell, 2005), and optimal psychological health (Grawitch et al., 2006). In the process of organizational change, employee recognition could help maintain the smooth continuation of the organization (Sghari, 2016). However, these studies mainly focus on the influence of leaders’ recognition on recipients while ignoring its possible influence on bystanders.

It is essential to explore third-party employees’ reactions to witnessing leaders’ recognition of colleagues, as nearly everyone cares about and refers to the lives of others for cognitive evaluation (Grienberger et al., 1997; O’Reilly et al., 2016). For example, abusive supervision of the coworker can motivate third-party affective, cognitive, and behavioral reactions (Mitchell et al., 2015). Third parties are likely to be angry about others’ experiences of injustice (O’Reilly et al., 2016). It is also found that the rude behavior witnessed by employees as a third party in the morning reduced their task performance throughout the working day (Woolum et al., 2017). When managers implement leadership behaviors, it is not only the targets who are the direct receivers of leadership behavior who will be affected but also involves others. Present studies have shown that witnesses would also be influenced by what they witness. Therefore, it is also very important whether and how employees react when they witness their colleagues being recognized by leaders as a third party. Unfortunately, the existing literature does not clearly reveal this point. Therefore, our research considers how the witness would think and act when they encounter how their colleagues are treated by their managers is a different and interesting perspective.

This study aims to explore the chain effect of witnessed employee recognition. Based on the Social Cognitive Theory (SCT) and Affective Events Theory (AET), people are influenced by others and the environment. Employees will be affected by the affective events they experience in the workplace. When employees witness or encounter certain workplace events, he/she would have reactions to these events (Bandura, 1986; Weiss and Cropanzano, 1996). Employee recognition is an evaluation result presented to employees. When an employee encounters a colleague recognized by their manager, there is a signal that the recognized one who creates value and contributes to the organization would be respected and appreciated by the leaders. The witness would also make their judgments about whether the managers conduct recognition rightly and fairly. That is to say, witnessed employee recognition might indicate the witness’s perceived organizational justice. Furthermore, organizational justice is a critical predictor of workplace wellbeing and work engagement. Considering the cognitive and behavioral reactions of witnesses, a model is developed to demonstrate the chain mediating effect while an employee observes colleagues get recognized.

Our theoretical model and research design allow us to make several meaningful contributions to employee recognition and related literature. First, we take the perspective of bystanders to explore the spillover effect of leadership, enriching the research on employee recognition. Recognition from leaders may have an expanding impact on employees in the organization, not only affecting the recognized employees but also those who witnessed the recognition. Second, our study contributes to the current knowledge on the benefits of witness leaders’ recognition of colleagues on perceived organizational justice and workplace wellbeing. Based on SCT and AET, we construct a chain intermediary model to explore the influence of witnessing others being recognized by leaders on employees’ own work engagement. When an employee sees that the leader recognizes his colleagues, it will affect his perception of organizational fairness and, further, affect his wellbeing and work engagement. That is to say, we aim to contribute to this research to explore how witness leaders’ recognition of colleagues affects work engagement.

Finally, justice and wellbeing are very important indicators that affect employees’ work and life, which deepens the research in organizational behavior studies.

## 2. Theory and hypothesis

The foundation of SCT is that human activities are determined by the interaction of three factors: individual behavior, individual cognition and other individual characteristics, and individual external environment (Bandura, 2001). Overall, man is not only the shaper of the environment but also the product of the environment. Based on SCT, employees would respond to the organizational environment by watching and learning from others, including their leaders and colleagues (Bandura, 1997). Affective Events Theory (AET) further explains that employees will be affected by affective events in the workplace, generate corresponding emotional reactions, and lead to long-term results. When witnessing employee recognition, this could be a trigger that makes the witness do reactions (Weiss and Cropanzano, 1996). Employee recognition encounter is not a strong event, so the employees might have transient emotional and cognitive reactions. When an employee gets recognized, it means that his/her dedication and value are approved by the organization or the manager. Employees might cognitively interpret this event as justice is guaranteed in the organization. Emotionally, recognition and justice enrich the positive experiences in the workplace, thus enhancing workplace wellbeing. In addition, good employees getting recognition inspires them to learn and imitate. They might take action to work harder and perform better.

### 2.1. Witnessed employee recognition

Because of the convenience and low cost, employee recognition is a very effective and popular leadership tool in management practice. Managers can make evaluations and approvals based on timely employees’ behavior. Compared with other incentive methods, it is more of a symbolic and commemorative material grant (John, 2010), such as awards to outstanding employees and thank-you letters from leaders. The ways of employee recognition are often non-monetary, with various formal or informal approaches. No matter what specific measures are adopted or certain forms of implementation, employee recognition is always based on the value created by employees for the organization.

Usually, better employees get recognized. Recognizing means the “good soldiers” of the organization are picked up after the screening and assessment based on the united standards. Thus, it is about organizational justice (Lance Frazier et al., 2010). Perceived organizational justice refers to anyone’s subjective perceptions of the fairness of allocations. Our research focuses on the process of interpersonal interaction between leaders and employees, as well as employees’ emotional responses and behavior choices while they watch employee recognition. We finally adopted a four-dimensional justice concept, namely, organizational justice (Colquitt, 2001), which includes distribution justice (employees’ perception of distribution results), procedural justice (employees’ recognition of distribution procedures), interpersonal justice (employees’ perceptions of the quality of leadership interactions), and informational justice (employees’ evaluation of leaders’ interpretation of distribution results and distribution rules).

Previous studies have confirmed that healthy LMX helps employees to perceive organizational justice (Shkoler et al., 2021). Employees get recognized by leaders for their excellent work performance, which shows the positive interaction between leaders and employees, and makes employees feel that leaders will attach importance to their work performance and achievements. When employees encounter that their colleagues are recognized by the leader because of their excellent work performance, dedication to the organization, or integrity, they are aware that the one who does a better job could get preferential treatment, which indicates that the organization is fair in reward distribution. The organization can effectively perform an appraisal of employees, which helps employees perceive organizational justice (Russell et al., 2007). Based on the SCT and AET, since employees witness their leader recognize the colleagues who perform well, they might believe that valuable workers will be treated fairly. This event could trigger emotional or behavioral reactions. The behavior of leader recognition sends a signal to employees that if they perform well, they can also be recognized and praised by the organization. Because of this belief, employee recognition encounters could strengthen witnesses’ perceived higher level of organizational justice.

Work engagement is a positive work-related state of fulfillment that is characterized by vigor, dedication, and absorption (Kahn, 1990). Employee recognition encounters may facilitate work engagement in several ways. (1) The positive praise and recognition of the leaders to their colleagues show that the leaders pay attention to the employees, to what they do, and to what they achieve. Through such behavioral interaction, leaders give positive feedback to the employees (Abugre and Sarwar, 2013), provide support for the employees’ work (Cheng et al., 2022), show respect for the employees’ work achievements (Zhao et al., 2022), and enable the employees to feel positive value and significance of work, thus increasing their work engagement. (2) By rewarding and praising outstanding employees, leaders convey the expected behavior and results of the organization to the members, so that employees can realize that hard work, team dedication, and other behaviors beneficial to the organization will be recognized (Xenikou, 2017). The recognized ones make fine examples in the workplace. According to SCT, people would adjust their behaviors to adapt to the environment by observing and learning from others. The recognized employees provide a reference for their actions. Once they witness employee recognition, they would enhance the impression that those who do a good job would be recognized. (3) When leaders can publicly commend employees’ achievements and contributions, it shows that employees can trust leaders to respect their labor income so that employees can get a sense of psychological security at work (Liu D. et al., 2022) and can have energy and ability to work. Recognizing the efforts of employees and praising their success and achievements will not only make employees close to and trust the organization but also effectively stimulate their enthusiasm and passion for work tasks (van Woerkom et al., 2020). All above, when witnessing employee recognition, the employees might be inclined to learn from them to work harder, behave better, and perform more. Therefore, the hypothesis is suggested as follows:

*Hypothesis 1*: Employees who witness employee recognition will (a) perceive more organizational justice and (b) be more engaged in work.

### 2.2. Mediation of perceived organizational justice

Previous studies have proved that justice perception is quite an important indicator that has a lot to do with human being’s life and work, such as individuals’ physical and mental health, life satisfaction, and work enthusiasm (Joshanloo et al., 2018). In a word, justice is positively related to a better life and satisfying work.

Wellbeing could be both physical and emotional. We focus on the emotional aspect. In general, wellbeing is a kind of people’s emotional experience and feeling in interpersonal communication or interaction process (Häusser et al., 2010; Schmitt et al., 2014). In this study, we pay close attention to the workplace environment and events. General wellbeing is much broader for this topic. Workplace wellbeing, which refers to an employee’s overall cognition and perception of satisfaction at work and the emotional and psychological experience and health status expressed at workplaces (Zheng et al., 2015; Li et al., 2021), is a more concrete and suitable variable according to our research design. In the existing study of wellbeing, organizational justice is an inseparable concept (Cassar and Buttigieg, 2015).

In the above discussion, we believe that when employees encounter their leaders recognize excellent colleagues, they can improve their perception of organizational justice. Based on the social cognitive theory, employee recognition conveys that the organization can fairly evaluate employees and reward outstanding employees. Previous studies have confirmed that the concept of organizational justice is related to a series of positive results (Pan et al., 2018). Through employee recognition, leaders can create a fair organizational atmosphere for employees, strengthen employees’ sense of work security, and enable them to deal with problems in the workplace positively and confidently, so that they can improve workplace satisfaction (Lee et al., 2021). In other words, witnessing employee recognition can promote employees’ workplace wellbeing by improving their perception of organizational justice. On the contrary, organizational justice is not only positively related to employee wellbeing but also positively related to other work aspects (De Angelis et al., 2021). When the perceived organizational justice is high, it indicates that employees can trust the organization’s distribution procedures, believe in the fairness of the distribution results and that the leadership’s interpersonal interaction and interpretation of the distribution are reliable and convincing (Lance Frazier et al., 2010). Under such circumstances, employees realize that a fair organization can reward their efforts and achievements. A fair organization can effectively reduce employees’ psychological stress, anxiety, pain, and other negative emotions (Lee et al., 2021), which gives them more mental resources to focus on work, so that employees are more loyal (Kaabomeir and Naami, 2016) and more committed (Rehman et al., 2021) to the organization, thus ultimately help improve employees’ work willingness and ability. Research on work engagement highlighted that people need three psychological preconditions: a sense of meaning, a sense of security, and availability. While employees get a higher organizational justice perception, the three psychological preconditions are provided. That is, the witness of employee recognition can promote employees’ work engagement by enabling them to gain a higher level of perception of organizational justice.

Therefore, the hypothesis is suggested as follows:

*Hypothesis 2*: Perceived organizational justice will mediate the relationship between employee recognition encounters with (a) workplace wellbeing and (b) work engagement.

### 2.3. Chain mediating effect

In the discussion of hypotheses 1, 2, when the employees encounter colleagues who get recognized, they are much more likely to perceive a high level of organizational justice and to feel happy. Workplace wellbeing turns out to be an indispensable factor to work outcomes and job performance in the management practice (Boncquet et al., 2020). Wellbeing can reduce employees’ job burnout and improve their work efficiency, so happy employees usually have better performance and lower turnover rates (Wright and Cropanzano, 2004; Spreitzer and Porath, 2012). The work engagement concept includes three dimensions, namely physiology, cognition, and emotion (Kahn, 1990). While employees are in a wellbeing state, the workplace psychological safety is strengthened, the organizational commitment is enhanced (Bakker, 2015; Yadav et al., 2022), they will feel that their work is intentional, and they are trusted and supported by leaders, which could make them feel confident and incompetent (Liu P. et al., 2022); thus, the goals they pursue can be achieved. Then, the three psychological preconditions for entering the state of work engagement are met. That is, justice perception and workplace wellbeing enable employees to obtain sufficient psychological resources to be objectively capable of getting into a state of high-level work engagement (Lee, 2015). That is, the witnesses who perceived a high level of organizational justice would likely feel happy (Loi et al., 2009) and be able to put much more effort to work.

Based on the above discussion, and combined with hypotheses 1 and 2, employee recognition encounter positively predict perceived organizational justice, which has a positive relationship with workplace wellbeing, and finally leads to higher work engagement. The hypothesis can be proposed as follows:

*Hypothesis 3*: Perceived organizational justice and workplace wellbeing will have a chain-mediating effect in the path of employee recognition encounter and work engagement.

**Figure 1:**
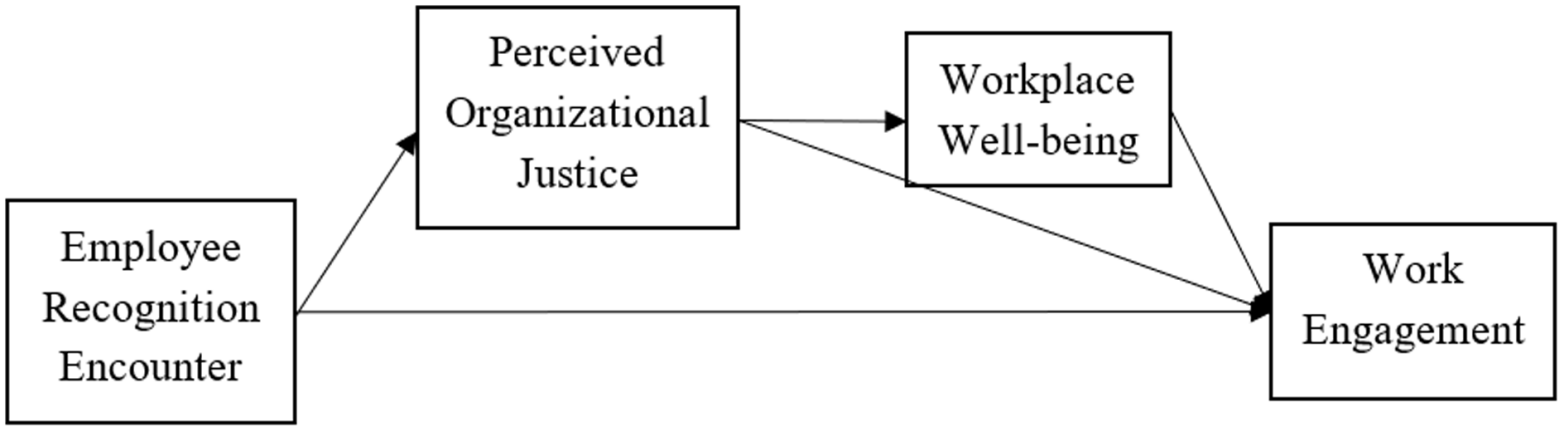
As proposed above, the basis hypotheses are showed on this figure.

The proposed research model is shown in Figure 1.

## 3. Methods

### 3.1. Sample and procedure

We used empirical research for this study, where we surveyed participants once a week for a month. Several days before the survey officially started, we asked the participants to complete a demographics survey. For the first time, on a Friday, we sent the survey that included the employee recognition encounter measure (T1). The second time was 7 days later; the next Friday, we sent the participants another survey that included perceived organizational justice measures (T2). And on the following Friday, we sent the participants a wellbeing survey (T3). On the fourth Friday, we asked the participants to complete the work engagement survey (T4). At each survey, we verified a 7-day timestamp.

In total, 500 questionnaires were collected. The participants come from finance, real estate, manufacturing, and other industries in the eastern coastal areas of China. After sorting and screening the questionnaires, some invalid questionnaires were eliminated. Finally, we got 258 valid questionnaires with an effective recovery rate of 51.60%.

In the final sample collected, there were 118 men (45.74%) and 140 women (64.26%); 120 (46.51%) were unmarried, and 138 (53.49%) were married, with an average age of 29.52 years (SD = 5.33). The average working experience is 6.18 years (SD = 5.35), and the average working time with current leaders is 2.13 years (SD = 2.01). Participants have diverse jobs, including bank staff, industry researchers, engineers, account managers, trainers, and financial personnel.

### 3.2. Measures

#### 3.2.1. Employee recognition encounter

Due to the limitations of current research, the development of employee recognition scale is under development. In the existing research, some scholars manipulated the variable of employee recognition through experiments; and some researchers used interviews with company HR to judge the employee recognition status of the surveyed subjects. Based on the widely accepted concept of employee recognition from Brun and Dugas (2008), we refer to other measurement methods of similar concepts and adopt the related items of the Contingent Rewards Leadership Behavior Scale on employee recognition behavior from Walumbwa et al. (2008). Contingent reward transactional (CRT) leader behavior refers to leader behaviors emphasizing the clarifying role and task requirements and providing followers with material or psychological rewards contingent on the fulfillment of contractual obligations (Walumbwa et al., 2008). That is quite close to the employee recognition implementation. We adopted five items from the CRLBS. We make a little adaptation to capture our study variable Employee Recognition Encounter by asking participants how often they see their managers perform employee recognition. The scale uses Likert’s five-point scale to measure, and the score ranges from 1 to 5, indicating “never” to “always.” Internal consistency reliability (Cronbach’s α) is 0.89.

#### 3.2.2. Perceived organizational justice

According to the research questions of this study, this article adopts the concept of a four-dimensional organizational justice defined by Colquitt (2001). The four-dimensional concept could better capture all the justice issues in the workplace. Each dimension has six, six, six, and four measurement items, for a total of 22 measurement items. Using Likert’s five-point scale, the score ranges from 1 to 5, which means “strongly disagree” to “strongly agree.” Internal consistency reliability (Cronbach’s α) is 0.91.

#### 3.2.3. Workplace wellbeing

The scale is adopted from the measurement of the dimensions of employee wellbeing in the “Employee well-being in organizations: Theoretical model, scale development, and cross-cultural validation” developed by Liang (2014), with a total of six items. This measurement scale takes cultural differences into consideration, which could better adapt to the Chinese context. Using Likert’s seven-point scale, the score ranges from 1 to 7, which means “strongly disagree” to “strongly agree.” Internal consistency reliability (Cronbach’s α) is 0.92.

#### 3.2.4. Work engagement

The Utrecht Work Engagement Scale (UWES), developed by Schaufeli and Bakker (2004), is a refined scale of work engagement measurement widely recognized and adopted in organizational behavioral studies. This scale version is short and clear, which is helpful for improving the fulfillment quality of questionnaires. The scale measures three dimensions, namely vitality, dedication, and concentration. Each dimension consists of three measurement items and a total of nine measurement items. Using Likert’s seven-point scale, the score ranges from 0 to 6 points, which means “not at all” to “always.” Internal consistency reliability (Cronbach’s α) is 0.91.

#### 3.2.5. Control variables

This study selected the participants’ gender, age, educational background, and work tenure as control variables to obtain more accurate results. Extant literature shows that demographic variables, like age, gender, and education have a significant impact on well-being, justice, and work motivation. (Latta and Fait, 2016; Cropanzano et al., 2017; Le et al., 2020). Meanwhile, with reference to many third-party studies (Woolum et al., 2017; Liang et al., 2022; Liu D. et al., 2022), gender, age, educational background, and work tenure were chosen.

### 3.3. Analysis strategy

According to the theoretical model proposed by this study, we need to further test the chain-mediating effect. So, we adopted the PROCESS macro module developed by Preacher and Hayes (2004): set the bootstrap resampling to 5,000 times, test the conditional indirect effect, and construct a 95% unbiased correction confidence interval. This method is based on the path analysis method and the least square method. Through calculation, the non-standardized path coefficient, standard deviation, and confidence interval are finally output.

## 4. Results

We estimated the reliability of each construct by calculating Cronbach’s α. The reliability values for all constructs range from 0.89 to 0.92, which are greater than the threshold of 0.70, thus demonstrating a high degree of internal consistency. Before the hypothesis test, we conducted a confirmatory factor analysis (CFA) to test the unidimensionality of the measurement models. The results show that the model fits well, the ratio of chi-square to a degree of freedom = 1.63, comparative fit index [CFI] = 0.92, Tucker–Lewis index [TLI] = 0.93, and root-mean-square error [RMSEA] = 0.05. CFA should be Table 1.

**Table 1:** CFA.

Model	χ^2^/df	CFI	TLI	RMSEA	SRMR
Model 4	1.63	0.92	0.93	0.05	0.05
Model 3	4.01	0.81	0.79	0.07	0.08
Model 2	5.37	0.66	0.71	0.09	0.10
Model 1	8.69	0.34	0.33	0.10	0.14

Model 1: employee recognition encounter + perceived organizational justice + workplace wellbeing + works engagement.

Model 2: employee recognition encounter + work engagement; perceived organizational justice + workplace wellbeing.

Model 3: employee recognition encounter; perceived organizational justice + workplace wellbeing; work engagement.

Model 4: employee recognition encounter; perceived organizational justice; workplace wellbeing; work engagement.

Means, standard deviations, and the intercorrelation among the variables are reported in Table 2. Employee recognition encounter was positively related to perceived organizational justice and work engagement. Perceived organizational justice was positively related to workplace wellbeing and work engagement. Intercorrelation analysis provided us with preliminary evidence of the relationship between variables and provided support for subsequent tests.

**Table 2:** Means, SDs, and inter-correlations among study variables.

Variable	Mean	SD	Gender	Age	WT	EB	ERE	POJ	WWB
Gender	1.54	0.57							
Age	29.53	5.33	0.00						
WT	6.18	5.35	0.07	0.02^*^					
EB	2.78	0.66	−0.03	−0.01	−0.00				
ERE	3.15	0.53	0.00	−0.00	0.02	0.01^*^			
POJ	3.88	0.64	0.01	−0.03	−0.01	−0.00	0.27^**^		
WWB	3.62	0.74	0.00^*^	0.01^*^	0.03	0.02^*^	0.15^*^	0.27^*^	
WE	3.79	0.88	0.01^*^	0.00	0.02^*^	0.03	0.33^**^	0.38^**^	0.47^**^

^*^p < 0.050, ^**^p < 0.010. To be shortened, WT, work tenure; EB, education background; ERE, employee recognition encounter; POJ, perceived organizational justice; WWB, workplace wellbeing; and WE, work engagement.

The results of the direct and indirect analysis are shown in Tables 3, 4. Hypothesis 1 supposed that employees who witness colleagues get recognized will be more engaged in work and perceive higher organizational justice. The results in Table 3 showed that at the 95% confidence level, employee recognition encounter was positively associated with work engagement (B = 0.101, CI [0.015, 0.202], excluding 0) and perceived organizational justice (B = 0.042, CI [0.003, 0.122], excluding 0). Therefore, hypothesis 1 was supported.

**Table 3:** Direct effect test.

Variable	B	Boot SE	95% confidence level	
			Lower	Upper
POJ	0.042	0.044	0.003	0.122
WE	0.101	0.039	0.015	0.202

*N* = 258. To be shortened, POJ, perceived organizational justice; WE, work engagement.

**Table 4:** Indirect effect test.

Pathway	B	Boot SE	95% confidence level	
			Lower	Upper
ERE → POJ → WWB	0.063	0.063	0.072	0.451
ERE → POJ → WE	0.025	0.059	0.027	0.032
ERE → POJ → WWB → WE	0.081	0.032	0.005	0.206
Total Indirect effect	0.117	0.131	0.153	0.5060

*N* = 258. To be shortened, ERE, employee recognition encounter; POJ, perceived organizational justice; WWB, workplace wellbeing; and WE, work engagement.

Hypothesis 2 predicted that perceived organizational justice would mediate the positive relationship between employee recognition encounter with workplace wellbeing and work engagement. The results reported in Table 4 showed that at the 95% confidence level, the mediating effect of perceived organizational justice in the path of employee recognition encounter and workplace wellbeing was significant, B = 0.063, and the CI is [0.072, 0.451], excluding 0. Moreover, it was significant in the path of employee recognition encounter and work engagement, B = 0.025, and the CI is [0.027, 0.032], excluding 0. Therefore, hypothesis 2 was supported. Hypothesis 3 proposed that perceived organizational justice and workplace wellbeing had a chain-mediating effect in the path of employee recognition encounter and work engagement. As presented in Table 4, the bootstrapping results showed that under the 95% confidence level, B = 0.081, and the CI is [0.005, 0.206], excluding 0. Therefore, hypothesis 3 got supported.

## 5. Discussion

Employee recognition is widely used in management practice (Garr, 2012). Managers can recognize and praise employees, their work behaviors, and results through oral praise, thank-you letters, emails, etc. From that, we can see employee recognition is very helpful in stimulating employees’ positive emotions and work behaviors with extremely low or even zero economic costs. The existing literature has proved that employee recognition is negatively related to negative organizational outcomes and positive to good ones. Recognized employees have the disposition to reduce withdrawal and counterproductive work behaviors, and their wellbeing and sense of organizational belonging improved. Thus, they tend to have a higher organizational commitment and be more satisfied with their jobs (John, 2010; Bradler et al., 2016). Our research found that employee recognition, as an important means of leadership practice, not only has a positive impact on recognized employees but also on bystanders.

Taking SCT and AET as the theoretical base, this study tries to clarify the chain-mediating effect between employee recognition encounter and work engagement *via* perceived organizational justice and workplace wellbeing. Adopting the empirical research method, 258 samples are collected. The results turn out that employees who witness employee recognition are inclined to have a higher organizational justice perception and, thus, gain more workplace wellbeing. By watching and learning from the recognized ones, employees would like to take them as fine examples at work and learn from them to deal with workplace issues. Therefore, employee recognition encounters could inspire witnesses to perceive more organizational justice, feel happiness, and engage more in work.

First, previous studies have confirmed that employee recognition is a very effective management tool that can promote better employee performance. Based on previous studies, our research found that employee recognition not only directly promotes recognized employees (Garr, 2012) but also has a positive effect on the bystanders who witness the recognition, enabling them to perceive better organizational justice, enhance their sense of wellbeing at work (Woznyj et al., 2021), and thus, healthy work environment and happiness fulfillment effectively improve employees’ performance while they have much confidence, energy, and psychological safety (Abuelhassan and Algassim, 2022). The results show that employee recognition is also beneficial to shaping a fair organizational atmosphere. We believe that positive leadership behavior can make employees feel the equality, respect, and support of the organization (Abugre and Sarwar, 2013), make employees happier, work harder (Zhao et al., 2022), and achieve the mutually beneficial development of the organization and employees.

Second, the previous third-party perspective on leadership has confirmed that leadership behavior has spillover effects. For example, research on abusive leaders found that if employees witness leaders abusing their colleagues, they would have angry emotional reactions, have an unfair perception of the organization (O’Reilly et al., 2016), and even they would imitate leaders, becoming more abusive and unfriendly. When employees witness the workplace incivility of leaders, they will feel that they are outsiders of the organization (Liu P. et al., 2022), which will bring more negative effects in the long run. The organization is a closed system. The leadership behavior conveys certain signals, and the employees will interpret such information and respond to it (Woolum et al., 2017). Previous bystander studies focus on negative behaviors in the workplace, discussing whether employees and leaders would choose to intervene, follow up on behaviors, and the reasons for their reactions to wrongdoings (Hershcovis et al., 2017). On the one hand, our research conclusion is consistent with previous research to some extent, which proves that third-party employees will respond to the leadership events they see and hear. On the other hand, we introduce positive leadership behavior’s bystander effect, which helps good management practice to be better. These findings also show that leaders must consider the objects, methods, and occasions of implementation when taking certain leadership behaviors. The relationship and interaction between leaders and employees in the organization may be closer and deeper than we usually think.

### 5.1. Theoretical and practical contributions

There are several important theoretical implications of our findings. First, we introduce a new viewpoint of employee recognition, bystander or witness. The mainstream employee recognition literature focuses on its direct receiver, exploring the interaction process and consequences. There is no doubt that employee recognition is a very effective leadership tool that helps to encourage and motivate employees. Nevertheless, the witness could be affected. The previous study has found that the recognition of center employees in the team has a positive effect on the performance of the entire team (Li et al., 2016). We take the witness’s perspective to see what they would feel and act while employees witness colleagues getting recognized. Second, based on the Social Cognitive Theory and Affective Events Theory, we develop a chain effect model that reveals how witnessing employee recognition contributes to work engagement. Plenty of research results have confirmed that there is a positive relationship between witness employee recognition and job performance. Our study expands this spillover effect that by watching recognition, the witnesses could also be inspired. They are inclined to perceive higher-level organizational justice, developing workplace wellbeing, and thus engaging in work. In this regard, our study enriches the justice and workplace wellbeing literature by introducing them as the chain-mediating role. Organizational justice and workplace wellbeing are both critical factors that do good to employees and then to organizations.

In addition to the theoretical significance discussed above, the results of our study have several important practical implications for managers and organizations. First, managers should recognize and appreciate employees who perform well, especially in public. Our research shows that taking time to give recognition is not simply a meaningless management activity but also an activity that can promote the wellbeing of employees. Leaders’ immediate employee recognition is not only beneficial to employees’ own performance but also to witnesses’ performance, which can effectively improve their wellbeing and work engagement. Second, leaders’ employee recognition should show fairness. It is found that leaders’ recognition will further affect employees’ wellbeing and work engagement through employees’ perceived organizational justice, so it is very important for leaders to be fair. Finally, managers should pay attention to employees’ wellbeing, which can effectively improve employees’ work engagement. Organizations and managers should take action to create good working conditions and inspire employees to work happily, gain self-esteem, and feel the value and meaning of work, such as by praising employees’ proactive behavior, rewarding their efforts, and giving positive feedback when they deserve it. This is beneficial to the organization, with an extremely low financial cost, to increase employees’ emotional commitment and sense of belonging to the organization and turn it into practical actions to enhance the competitiveness of the organization.

### 5.2. Limitations and future directions

Despite the abovementioned contributions, the current study is subject to several limitations. First, the measurement scale of employee recognition is not mature yet. The measurement tools used in this study are from the relevant items in the Contingent Reward Leadership Behavior Scale of Walumbwa et al., (2008). Although the scale has passed a good reliability and validity test, it is still in good need that researchers can develop a more scientific and reasonable concept and compile a clearly defined employee recognition measurement scale in the future. During the data collection, we did not measure the variable change at different times, which could face the challenge of robust testing. Therefore, that makes the results lack credibility and reliability. One more issue about the data is although we have adopted a 7-day interval method to collect the data, the measurement variables are self-reported from the same participants, and the common method biases are still an unsolved problem. Maybe, other data methods can be used, such as the leader–employee matching method, diary method, and even experimental design. Regarding control variables, we just collected demographic variables. As a matter of fact, there are many other variables related to our research results, such as bystanders’ personalities (Liu D. et al., 2022) and power (Hershcovis et al., 2017). It will be more solid if the study makes a thorough consideration of control variables. Nevertheless, we invite future research to replicate our findings using other methods to validate the results reported here.

Another limitation of this study is that we introduce two mediators, perceived organizational justice and workplace wellbeing. There might be more factors that could implement on this path and cause different effects. Some studies argue that recognition may cause jealousy and dissatisfaction and ultimately may hurt the team results (Pearsall et al., 2010). Followed by this issue, different people have different reactions to the same event. For employees who have various personalities and characteristics, there are possibly varied reactions. These are interesting research directions for future researchers to do intensive studies further. There is a very important challenge in our theoretical model. We propose a chain-mediating effect, in which we argue that witnessing employee recognition is positively related to higher organizational justice perception, leads to more workplace wellbeing, and enables employees to engage more in work. However, there are other plausible orders. A study has argued that psychological health problems influence perceptions of organizational justice (Lang et al., 2011). It could be another reasonable explanation that witnessing employee recognition might be more directly linked to work engagement, which in turn leads to better wellbeing, and then higher perceived justice.

Moreover, this study mainly explains the mediating mechanism without considering the boundary condition. We believe that there are much more details to be revealed. The situation and form of employee recognition, such as whether it is in public or private, formal or informal, might change its functions. Moreover, the implied participants of employee recognition, the manager who acts out this leadership behavior, and the employees who receive this recognition, their interaction, and relation with the witnesses could also influence the whole effect. Exploring these effects deepens the understanding and broadens the scope of this topic. We are hopeful that this study will encourage future inquiry in this exciting area of research.

Another interesting and meaningful research direction is to compare the bystanders with the recognized colleagues. What if employees witness their leaders reward their colleagues who are inferior to them? This difference between good and bad might cause different reactions. Will they be envious? Will they be aware of the unfairness of the organization? This comparative study will enable us to have a deeper understanding of the relationship between leaders and employees, as well as between employees and employees.

### 5.3. Conclusion

Based on the Social Cognitive Theory and Affective Events Theory, this study develops a model from the witness perspective, investigating the thought and behavior when an employee witnessed colleagues get recognition. Previous studies have found that employee recognition is a widely used incentive implement that helps motivate employees to gain wellbeing, be more engaged in work (Bradler et al., 2016), mobilize employees’ active resources, and contribute to work performance (Merino and Privado, 2015). Our research adopts the empirical research method by asking the participants to do the four-time survey in a month, and 258 samples are collected. Using SPSS20.0 and its PROCESS macro module, hypotheses are tested. In this study, we find that employee recognition encounter has a significantly positive relationship with work engagement. That is, by watching employee recognition not received, leadership effectiveness also is achieved. To be specific, employee recognition encounters could improve employees’ perceived organizational justice, increase workplace wellbeing, help them actively engage in work, and thus facilitate the growth and development of the organization. Perceived organizational justice and workplace wellbeing have a chain-mediating effect in this pathway.

Therefore, leaders should be good at discovering the strengths and advantages of employees and be able to recognize and praise employees for their achievements and contributions to management practice. Adopting positive leadership behaviors and creating a sound working atmosphere and fair organizational environments, enable employees to work harder and work happier.

## Data availability statement

The raw data supporting the conclusions of this article will be made available by the authors, without undue reservation.

## Ethics statement

Ethical review and approval was not required for the study on human participants in accordance with the local legislation and institutional requirements. The patients/participants provided their written informed consent to participate in this study.

## Author contributions

TY: conceptualization, methodology, data curation, and formal analysis. TY and XJ: investigation, supervision, writing of original draft, and writing of review and editing. All authors contributed to the article and approved the submitted version.

## Funding

This study was partly supported by the National Natural Science Foundation Project of China, grant number 71472140.

## Conflict of Interest

The authors declare that the research was conducted in the absence of any commercial or financial relationships that could be construed as a potential conflict of interest.

## Publisher’s note

All claims expressed in this article are solely those of the authors and do not necessarily represent those of their affiliated organizations, or those of the publisher, the editors and the reviewers. Any product that may be evaluated in this article, or claim that may be made by its manufacturer, is not guaranteed or endorsed by the publisher.
